# On Amputation

**Published:** 1892-10-29

**Authors:** 


### NEWCASTLE-UPON-TYNE INFIRMARY.

### On Amputations.

The results of amputation have, in hospital practice, always been regarded as a fair test of the salubrity of the institution, and, perhaps, in no department of surgery is the contrast greater between the practice of to-day and that of, say, twenty years ago than in the method of treating an amputation wound, and the results now and then obtained. Twenty years ago, in the Newcastle-upon-Tyne Infirmary, forty per cent. of the major amputation cases died; and, now, taking a period of thirteen years and nine months, viz., from

April 1st, 1878, to December 21st, 1891, the mortality is only eight per cent. This is very striking, and the more so as the building is old and obsolete, and, in the opinion of the late Sir James Y. Simpson, the wards are quite unfitted for the successful treatment of wounds. The ill success, however, which attended amputations formerly was not due to the age of the building, nor to the construction of the wards, but to the treatment adopted. What was that treatment? Let us compare the method employed then with that in vogue to-day. There is a great deal of accidental surgery in a hospital situated like the Newcastle Royal Infirmary, and this kind of surgery, necessitating primary amputation, was much more fatal than that undertaken for disease. It is still more fatal, but not nearly to the same extent. During the period mentioned 272 amputations were performed for injury, with 35 deaths—12·9 per cent.—415 for disease, with 20 deaths, 4·8 per cent., making 687 cases in all, with 55 deaths. In the old days a man was admitted, let us say, requiring primary amputation of the thigh. His limb was washed with warm water and soap, if it were dirty, but if it were apparently clean the formality was not by any means always resorted to. Then Petit's or Signoroni's tourniquet was applied to the femoral artery. The surgeon might wash his hands before touching the limb, or he might not, and often he deferred the process till the operation was over, when, for obvious reasons, he washed his hands. The chloroform was given by a student. The instruments were laid out in a tray upon a towel. The house surgeon, who assisted the surgeon, might, and very often had, just come from making a post-mortem examination, his hands not free from odour, and, therefore, though thoroughly washed with hot water and soap, not by any means surgically clean. The methods of amputation were the same then as now; the limb removed, the vessels were sought for, seized with toothed forceps and tied with silk or hemp ligatures, which were left long and brought out at one end of the wound. Then the raw stump was sponged with sponges presumed to be clean, but not above suspicion, and well washed with cold water. Bleeding having ceased, sutures were introduced, not too closely together, of silk or wire, and the stump lightly dressed with lint, saturated with a mixture of linseed oil and carbolic acid. A splint was sometimes applied, but usually the stump was simply laid upon a pillow. As soon as the patient recovered from the chloroform an opiate was administered and a liberal allowance of whisky was given as a routine practice. Next day the wound was dressed, and before many days the stump was poulticed with linseed meal, tons of which were used for this purpose yearly. The stump suppurated, the poultices were changed every few hours, and on each occasion, however careful the nurses were, the process was painful and disturbing to the patient. In course of time the ligatures came away, sometimes they were gently pulled to hasten their separation, and often they did not separate for many anxious, weary days. Under this method of treatment hardly a single patient escaped an attack of surgical fever, secondary hæmorrhage was common, and forty per cent. of the patients died of pyæmia or some other form of blood poisoning. If they survived, the wound healed by granulation. There were daily dressings with lint, soaked with water or red lotion, covered with gutta-percha tissue. The flaps were drawn and strapped together with long strips of sticking-plaister, and from time to time the granulations were rubbed over with bluestone or nitrate of silver. No stump ever healed by first intention. Recovery, when it took place, was prolonged and tedious. Nothing could be more discouraging, and looking at the matter with our present views, nothing could be more natural and consequent. Had this, or any similar mortality, continued, large hospitals would, I am satisfied, not have continued to be used for amputations. The mortality in private practice and in small hospitals was very much less than in even the very best of the large establishments. Now the mortality in large hospitals is even lower than it is in the small cottage hospitals.

A patient who is admitted into the Newcastle Infirmary to-day for primary amputation, first has the limb shaved, washed and scrubbed with hot water, soap and a nail brush, washed with turpentine, spirits of wine, and lastly with a solution of perchloride of mercury. The limb is rendered bloodless by elevation, and maintained so by means of the indiarubber tourniquet. The instruments, thoroughly scrubbed and clean, are placed in a white porcelain dish in an antiseptic solution. Towels, wrung out of a similar fluid, are arranged about the limb and the body. The surgeon takes off his coat, and either dresses himself in a clean white coat, which completely covers all but his hands, face and feet, or tucks up his sleeves and puts on a clean long white linen apron. Having washed and scrubbed his hands with hot water and soap, and washed them first with turpentine and then in a solution of perchloride of mercury, he amputates. The vessels are seized with Spencer Wells' forceps and tied with aseptic silk, which is cut short. The cut surfaces are washed with a warm solution of corrosive sublimate and absolutely clean sponges. Some surgeons sprinkle freely upon the raw surfaces powdered boracic acid, and then the wound is closed, an indiarubber drainage tube being generally used, though the tendency is rather to dispense with this adjunct, and in such an amputation as the forearm of an adult, and arm of a child, it is often not used. The wound is closed very carefully and as accurately as possible, sometimes with a continuous suture of catgut, but generally with interrupted sutures of fishing gut. Some surgeons at this stage powder the wound with iodoform: then a piece of green protective is usually applied, a little antiseptic gauze, and over all a thick covering of dry absorbent cotton wool is firmly secured with bandages, and the stump fixed in a splint. The anæsthetic usually employed is chloroform or the A. C. E. mixture, occasionally ether is given, but whatever anæsthetic is used, it is administered by the special officer appointed for the purpose. An amputation now frequently heals by first intention, and dressings are infrequent, often there is only one, when the drainage tube is removed some twenty-four hours after the operation, and another. On the 5th of this month a muscular young labourer, all flesh and blood, was admitted requiring primary amputation of the thigh—just the sort of patient who, under the old system, would have been sure to have done badly. He was much collapsed, and there was little doubt he had sustained a laceration of the left kidney. The limb was removed at its lower third. No drainage tube was used. It was not dressed for a week, and it healed throughout without the secretion of a single drop of pus. Neither opium nor alcohol are given as formerly, simply because an amputation has been performed; both are sometimes required, but not nearly to the same extent as used to be the case under the old system, and often neither is found to be necessary. There is now no looking forward with dread to the changing of dressings—the process is a painless one when it is required. Secondary hæmorrhage is practically unknown, and any form or degree of blood poisoning rare and quite exceptional. The contrast between the two systems and their results is very apparent. It is possible in the future some of the precautions that are now taken may be discontinued, but it will be a long time before the two principles of asepticism, which we undoubtedly owe to Sir J. Lister, and dry, firmly bandaged dressing, infrequently changed, for which we are indebted to the late Mr. S. Gamgee, of Birmingham, are superseded.

